# Activation of the Wnt signaling pathway and its role in epithelial-mesenchymal transition and hepatic fibrosis in alveolar echinococcosis

**DOI:** 10.3389/fcimb.2025.1583802

**Published:** 2025-05-27

**Authors:** Binjie Wu, Yuxuan Yang, Shilei Cheng, Zhixin Wang, Haining Fan

**Affiliations:** ^1^ Research Center for High Altitude Medicine, Qinghai University, Key Laboratory of High Altitude Medicine (Ministry of Education), Key Laboratory of Application and Foundation for High Altitude Medicine Research in Qinghai Province (Qinghai-Utah Joint Research Key Lab for High Altitude Medicine), Laboratory for High Altitude Medicine of Qinghai Province, Xining, Qinghai, China; ^2^ Qinghai Research Key Laboratory for Echinococcus, Qinghai University, Xining, Qinghai, China; ^3^ Department of Hepatopancreatobiliary Surgery, Affiliated Hospital of Qinghai University, Xining, Qinghai, China

**Keywords:** DKK-1, epithelial-mesenchymal transition, hepatic alveolar echinococcosis, hepatic fibrosis, Wnt signaling pathway

## Abstract

**Objective:**

This study examined the effects of *Echinococcus multilocularis* infection on the activation of the Wnt signaling pathway in hepatocytes, its association with epithelial-mesenchymal transition (EMT), and its role in *E. multilocularis*-induced hepatic fibrosis.

**Methods:**

Hepatic lesion tissues were obtained from 20 patients with clinically diagnosed alveolar echinococcosis (AE). These tissues were categorized into near-lesion and distant-from-lesion groups. Additionally, a murine model of AE infection was developed through the injection of *E. multilocularis* protoscoleces. The mice were divided into control, Wnt pathway enhancement (Wnt3a), and Wnt pathway inhibition (DKK1) groups. Four weeks post-infection, AAV-EGFP, AAV-Wnt3a-EGFP, or AAV-DKK1-EGFP vectors were administered, followed by tissue collection four weeks later. Both human and murine liver tissues were analyzed using Masson’s trichrome, hematoxylin and eosin (H&E), and Sirius red staining, as well as immunohistochemical and western blot analyses to assess protein expression levels associated with EMT and fibrosis.

**Results:**

Elevated expression levels of Wnt3a, β-catenin, N-cadherin, Col1a1, α-SMA, Vimentin, CTGF, and TGF-β were observed in tissues adjacent to human AE lesions and in the Wnt3a-treated mouse group. Conversely, E-cadherin expression was low. Immunohistochemical analysis demonstrated lower expression of Wnt3a, β-catenin, and other EMT- and fibrosis-related proteins in perilesional areas in human tissues and in the DKK1-treated mouse group, while increased E-cadherin expression was elevated. Inflammatory cell infiltration and fibrosis were observed near human lesions, whereas the DKK1-treated mouse group exhibited significantly reduced fibrosis.

**Conclusion:**

The Wnt signaling pathway plays a key role in the development of hepatic fibrosis associated with AE infection. Its activation is positively correlated with EMT and the increased expression of fibrogenic markers, including Collagen I, CTGF, and TGF-β, thereby contributing to the progression of hepatic fibrosis in hepatic AE.

## 1 Introduction

Alveolar echinococcosis (AE) is a zoonotic parasitic disease caused by the larval stage of *Echinococcus multilocularis*. This infection primarily affects various organs and tissues, including the liver, lungs, brain, and bones (Wen et al., 2019). The liver is the most commonly affected organ, with nearly all reported cases exhibiting hepatic involvement (Peters et al., 2021). Hepatic fibrosis results from *E. multilocularis* infection in the liver, with alterations in the immune system and modifications to the intrahepatic microenvironment play a significant role. These promoted and stimulated the activation of hepatic stellate cells (HSCs). Once activated, HSCs leading to increased expression of alpha-smooth muscle actin (α-SMA) and collagen type 1 (Col1a1). These cells secrete substantial amounts of collagen, adhesion proteins, and amorphous matrix components, contributing to excessive extracellular matrix (ECM) deposition and fibrous tissue proliferation (Li et al., 2023).

In most chronic liver diseases, hepatic stellate cell activation and ECM deposition are associated with epithelial-mesenchymal transition (EMT) (Kong et al., 2020; Su et al., 2020; Kumar et al., 2021). The upregulation of α-SMA is widely recognized as both a key indicator of EMT and an essential contributor to fibrosis progression. During EMT, epithelial cell markers such as E-cadherin are downregulated, while mesenchymal markers, like N-cadherin and Vimentin, are upregulated. Fibrotic processes are often mediated by pro-fibrotic factors, notably transforming growth factor-β (TGF-β) and connective tissue growth factor (CTGF) (Chen et al., 2023; Trampuž et al., 2023). TGF-β enhances collagen synthesis by hepatic stellate cells, promoting significant ECM accumulation while inhibiting ECM and collagen degradation. CTGF promotes cell mitosis, differentiation, adhesion, and proliferation, in addition to regulating angiogenesis.

TGF-β, a growth factor involved in tissue repair, contributes to fibrosis by activating the downstream Wnt signaling pathway (Trampuž et al., 2023). The Wnt/β-catenin pathway has been implicated in fibrotic processes across various organs, including the lungs, kidneys, skin, and liver. Abnormal accumulation of β-catenin is closely related to hepatic stellate cell (HSCs) activation, epithelial-mesenchymal transformation (EMT) and excessive deposition of extracellular matrix (ECM) (Liu J. et al., 2022; Wang et al., 2024). Vimentin can anchor and support organelles within the cytoplasm of mesenchymal cells. During epithelial-mesenchymal transition (EMT), the expression of vimentin is upregulated. Cancer cells often exhibit features such as EMT during metastasis, and vimentin promotes EMT by altering cell shape and motility (Wang et al., 2017; Kong et al., 2020). Wnt proteins regulate β-catenin accumulation, which, upon reaching a threshold level, activates downstream target genes, promoting the development of hepatic fibrosis. Dickkopf-1 (DKK1), a member of the DKK protein family, functions as an extracellular inhibitor of the Wnt signaling pathway. Numerous studies have demonstrated that inhibition of Wnt/β-catenin signaling via DKK1 reduces hepatic stellate cell activation, slows fibrosis progression, and mitigates EMT in liver lesions in both *in vivo* and *in vitro* models (Wang et al., 2017; Liu QW. et al., 2022). Despite these findings, the role of this pathway in hepatic fibrosis associated with AE infection has not been fully investigated. This study aimed to assess whether EMT occurs in patients with AE infection and to determine if DKK1-mediated inhibition of intrahepatic Wnt/β-catenin signaling can attenuate EMT and reduce AE-induced hepatic fibrosis.

## 2 Materials and methods

### 2.1 Experimental animals and sample sources

Specific pathogen-free (SPF) C57 male mice (Charles River), aged 4 weeks and weighing 18–20 grams, were maintained under controlled conditions at 25°C with a 12-hour light/dark cycle. Standard rodent feed and water were provided. The *E. multilocularis* infection model was established via hepatic portal vein injection. Four weeks following infection, recombinant adeno-associated virus (AAV) vectors—AAV-EGFP, AAV-Wnt3a-EGFP, and AAV-DKK1-EGFP (Cyagen, Guangzhou, China)—were administered via tail vein injection. Each mouse was injected once with AAV vectors at a concentration of 5*10^11^vg/ml for 200ul. The mice were assigned into three groups, with five mice per group. All animal procedures received ethical approval for from the Qinghai University Affiliated Hospital (Project number P-SL-2019039). Treatment and euthanasia were conducted in accordance with ethical and humane guidelines.

Hepatic AE lesion specimens were obtained from 20 patients at Qinghai University Affiliated Hospital. The basic information of the patients is shown in **Supplementary Table S1**. Pathological examination confirmed all specimens as hepatic AE. The samples were categorized based on their proximity to the lesion: tissue located within 0.5 cm of the infiltrative margin (Close group) and hepatic parenchyma more than 2 cm from the lesion without visible involvement (Distant group). Liver specimens underwent hematoxylin and eosin (H&E) staining, Masson’s trichrome staining, immunohistochemical staining, and western blot analysis. Ethical approval for the study was granted by the Affiliated Hospital of Qinghai University (Project number P-SL-2019039) in compliance with the Declaration of Helsinki and the Declaration of Istanbul. Informed consent was obtained from all patients.

### 2.2 Isolation of *E. multilocularis* protoscoleces

*E. multilocularis* protoscoleces were isolated following previously described protocols (Liu et al., 2021). Echinococcal cyst tissue was harvested from the peritoneal cavities of breeding Mongolian gerbils obtained from the Qinghai Province Echinococcosis Laboratory. The tissue was dissected into small fragments in pre-cooled normal saline and filtered through sterile 100 μm and 40 μm filters to collect the protoscoleces. Continuous washing with normal saline was performed throughout the filtration process. Protoscoleces were separated by natural sedimentation and temporarily stored in 1640 culture medium supplemented with 15% fetal bovine serum. Following 24 hours of incubation at 37°C under normal conditions, microscopical examination confirmed the viability of the protoscoleces, which were then prepared for use in model establishment.

### 2.3 Establishment of *E. multilocularis* infection mouse model via hepatic portal vein injection

Mice in the *E. multilocularis* infection group were injected with 2,000 protoscoleces each following anesthesia with isoflurane. The procedure involved fasting the mice for 12 hours prior to surgery and withholding water for 2 hours before anesthesia. Preoperative body weight was recorded, and abdominal hair was removed to facilitate surgery and minimize the risk of postoperative infection.

Following approximately 10 minutes of anesthesia induction, a 3 cm midline abdominal incision was made to expose the hepatic portal vein near the liver. A well-mixed suspension containing 2,000 protoscoleces per 100 μL was injected into the hepatic portal vein using a 24G fine needle. An additional 100–200 μL of normal saline was administered to flush the needle, ensuring no residual protoscoleces remained, resulting in a total injection volume of 200–300 μL. Hemostasis was achieved by applying gentle compression with a cotton swab for 1–2 minutes after needle withdrawal. To compensate for fluid loss during surgery, a small volume of normal saline was injected into the peritoneal cavity.

The incision was closed using absorbable sutures and disinfected with iodophor. The mice were then placed inverted on a 37 °C-heating pad and monitored until full recovery, including the ability to turn over and move independently. Once stable, they were transferred to clean cages. Daily monitoring and care were provided for one week following the surgery to ensure recovery and well-being.

### 2.4 AAV-Wnt3a and AAV-DKK1 intervention in *E. multilocularis*-infected mice

Four weeks after model establishment, mice in the control group received tail vein injections of AAV-EGFP. The Wnt pathway activation group received AAV-Wnt3a-EGFP, while the Wnt pathway inhibition group received AAV-DKK1-EGFP. Following the injections, the mice were housed under identical environmental conditions.

Two weeks after the AAV injections, frozen liver sections were examined to assess the expression of green fluorescence. Additionally, small animal imaging was performed to confirm successful AAV delivery and hepatic localization.

### 2.5 Pathological staining of patient liver lesions and mouse liver tissues

Four weeks after AAV tail vein injection, the mice were euthanized, and liver tissues were collected and fixed in 4% paraformaldehyde. The fixed tissues were embedded in paraffin, sectioned into 4 μm slices, and deparaffinized with water. H&E staining was performed for histological evaluation.

Masson’s trichrome staining and Sirius red staining were conducted to assess collagen deposition. Five randomly selected areas from each sample were analyzed using ImageJ software to quantify the proportion of collagen fiber area. Immunohistochemical staining was performed by incubating the sections with primary antibodies against Wnt3/3a, β-catenin, E-cadherin, N-cadherin, Col1a1, α-SMA/Acta2, Vimentin, CTGF, and TGF-β (all diluted 1:2000, Abcam, Cambridge, UK) at 4°C for 12 hours. After washing, the sections were incubated with a secondary antibody using the UltraSensitive™ SP (Ms/Rb) IHC Kit (MXB, China) at 25°C for 10 minutes. Visualization was achieved with DAB, followed by hematoxylin counterstaining and mounting. Liver lesion tissues from patients underwent identical processing and staining procedures.

### 2.6 Western blot analysis of protein expression in human and mouse samples

Four weeks after AAV injection, mice were euthanized, and liver tissues were collected for protein extraction. Liver tissues from both patient and model mice were lysed using RIPA lysis buffer (Solarbio, China). A total of 15 μg of protein from each sample was mixed with loading buffer, boiled at 100°C for 10 minutes, and separated by 10% sodium dodecyl sulfate-polyacrylamide gel electrophoresis (SDS-PAGE). The separated proteins were transferred onto polyvinylidene difluoride (PVDF) membranes, which were then blocked with rapid protein-free blocking buffer (Solarbio, China) for 10 minutes.

Primary antibodies (diluted 1:1000, Abcam, Cambridge, UK) targeting Wnt3/3a, β-catenin, E-cadherin, N-cadherin, Col1a1, α-SMA/Acta2, Vimentin, CTGF, TGF-β, GAPDH, and β-actin were applied to the membranes. Incubation with the primary antibodies was carried out overnight at 4 °C. The membranes were subsequently incubated with HRP-conjugated goat anti-rabbit secondary antibody (1:5000, Proteintech, China) at 25 °C for 1 hour. Protein detection was conducted using ECL substrate (Proteintech, China). Band intensities were measured using ImageJ software and normalized to GAPDH and β-actin.

### 2.7 Study parameters

Immunohistochemical staining was performed to evaluate the expression of Wnt3a, β-catenin, E-cadherin, N-cadherin, Col1a1, α-SMA, Vimentin, CTGF, and TGF-β in liver tissues. Protein expression levels of these markers were measured using western blot analysis in both distant-from-lesion and near-lesion groups.

### 2.8 Statistical analysis

Statistical analyses were performed using GraphPad Prism 8, IBM SPSS Statistics V23.0, and RStudio. The Shapiro-Wilk test was used to assess data normality. Quantitative data were presented as mean ± standard deviation (*SD*), while enumeration data were presented as numbers or rates. Quantitative data with a normal distribution were analyzed using t-tests or one-way analysis of variance (ANOVA), and enumeration data were analyzed using the chi-squared test or Fisher’s exact test. All data sets satisfied the normality assumptions required for these parametric tests. A p-value of less than 0.05 was considered statistically significant.

## 3 Results

### 3.1 Changes in hepatic fibrosis, pathology, and related protein expression in patients with AE

**Figure 1:**
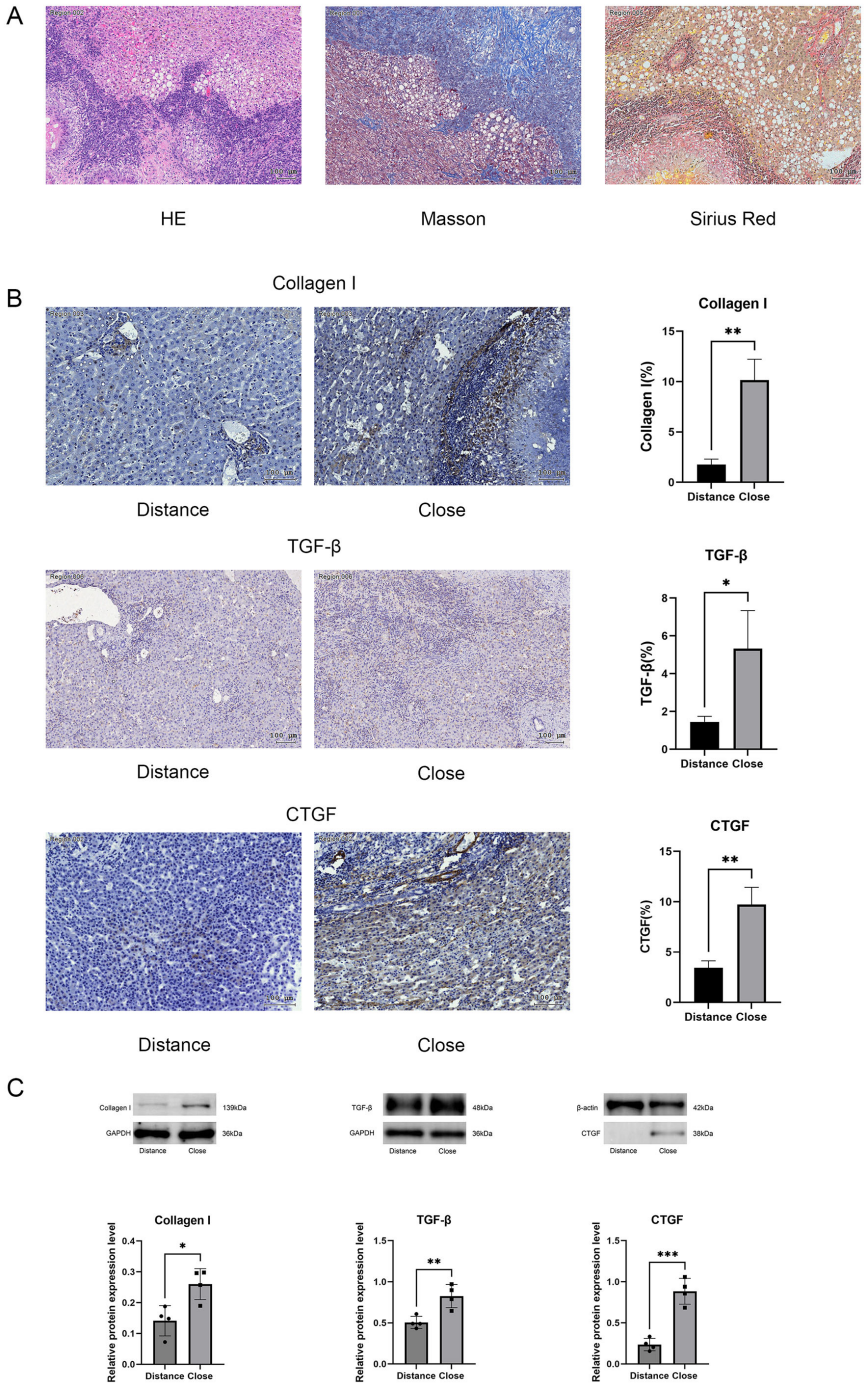
**(A)** Representative images of H&E, Masson’s trichrome, and Sirius red staining. **(B)** Immunohistochemical analysis comparing collagen type I, TGF-β, and CTGF expression in perilesional and distal hepatic tissues. **(C)** Western blot analysis of collagen type I, TGF-β, and CTGF protein expression, with relative quantification. Scale bar = 100 μm. Data are presented as mean ± *SD*. *p < 0.05, **p < 0.01, ***p < 0.001, ****p < 0.0001.

Histopathological analysis of liver tissue was performed using H&E, Masson’s trichrome, and Sirius red staining. The results of frozen liver sections and animal images are shown in the **Supplementary Figure S2**. H&E staining indicated extensive infiltration of inflammatory cells surrounding the lesions, tuberculoid caseous necrosis at the lesion center, and lipid vacuoles in the peripheral regions of the lesions (**Figure 1A**). Masson’s trichrome and Sirius red staining demonstrated extensive fibrosis around the lesions (**Figure 1A**).

Western blot analysis indicated significantly higher expression levels of type 1 collagen (Col1a1) in tissues near the lesions compared to those in distant-from-lesion areas (**Figure 1C**). Elevated expression of pro-fibrotic factors, including CTGF and TGF-β, was detected (**Figure 1C**). Immunohistochemical staining further confirmed that the expression of Col1a1, CTGF, and TGF-β was significantly stronger in near-lesion tissues than in distant tissues (**Figure 1B**).

### 3.2 EMT occurrence and related protein expression changes in liver tissues of patients with AE

**Figure 2:**
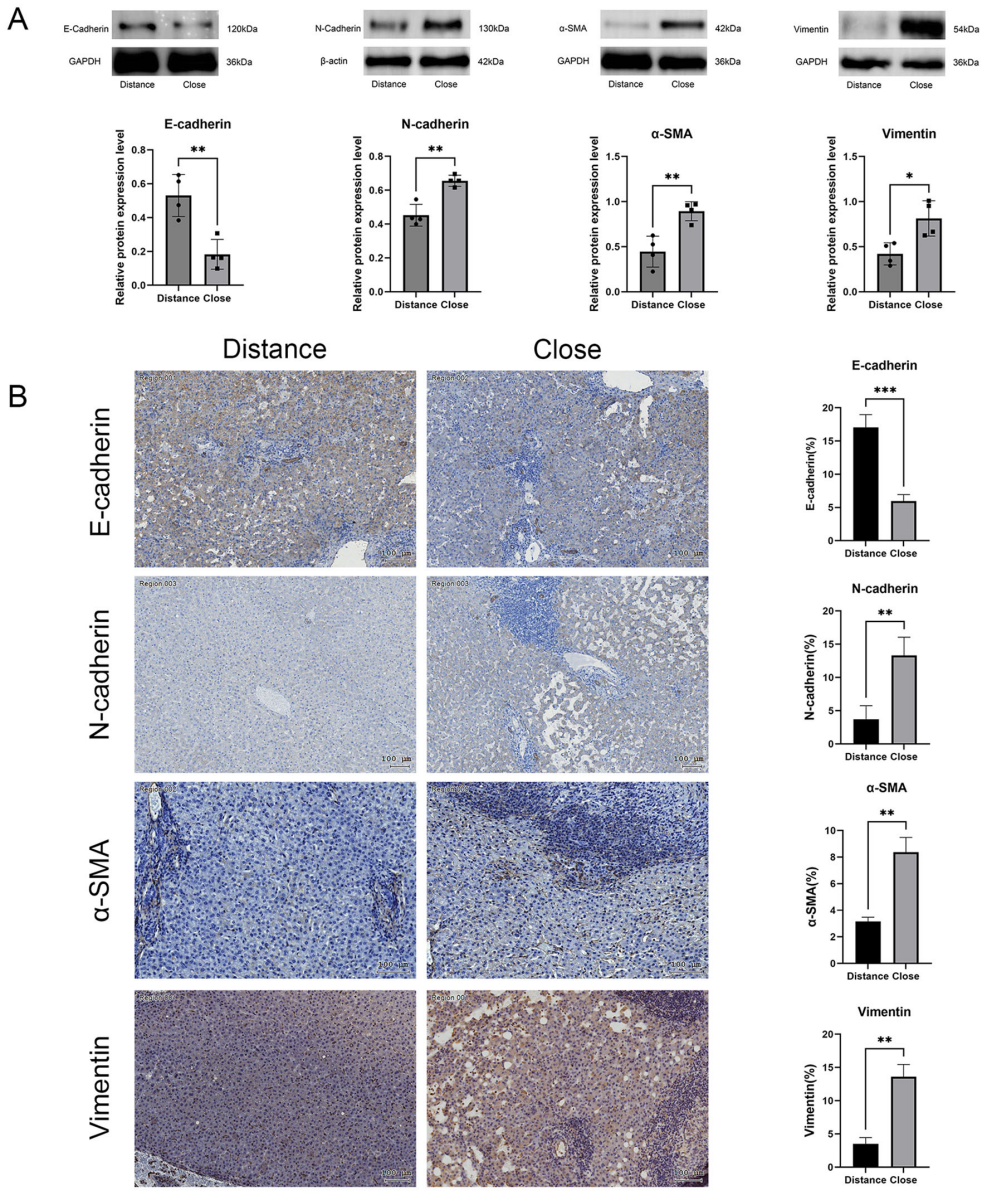
**(A)** Western blot analysis of E-cadherin, N-cadherin, α-SMA, and Vimentin protein expression, with relative quantification. **(B)** Immunohistochemical staining comparing E-cadherin, N-cadherin, α-SMA, and Vimentin expression in perilesional and distal hepatic tissues. Scale bar = 100 μm. Data are presented as mean ± *SD*. *p < 0.05, **p < 0.01, ***p < 0.001, ****p < 0.0001.

Western blot analysis of liver tissues from patients demonstrated a reduction in E-cadherin expression in near-lesion areas, while Vimentin, N-cadherin, and α-SMA expression were significantly elevated in these regions (**Figure 2A**). Immunohistochemical staining further demonstrated that E-cadherin expression was lower in near-lesion regions, whereas Vimentin, N-cadherin, and α-SMA expression were significantly increased (**Figure 2B**). These findings suggested that EMT occurred in the peripheral region of AE-infected human liver tissue.

### 3.3 Elevated Wnt3a and β-catenin expression in liver tissue of patients with AE

**Figure 3:**
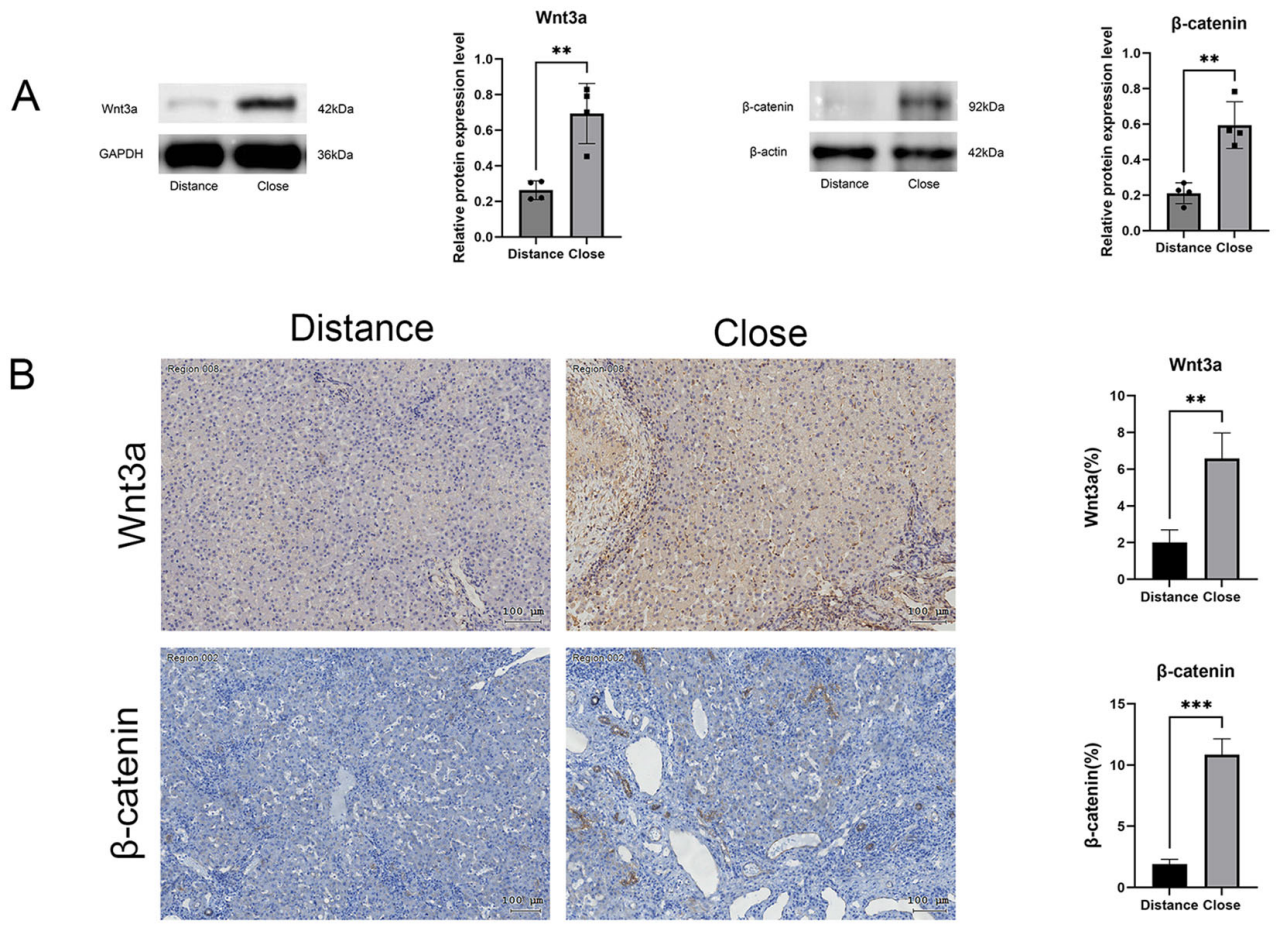
**(A)** Western blot analysis of Wnt3a and β-catenin protein expression, with relative quantification. **(B)** Immunohistochemical analysis quantifying positive staining areas in perilesional and distal hepatic tissues. Scale bar = 100 μm. Data are presented as mean ± SD. *p < 0.05, **p < 0.01, ***p < 0.001, ****p < 0.0001.

The Wnt/β-catenin signaling pathway plays a key role in hepatic fibrosis. Elevated protein expression levels of Wnt3a and β-catenin were observed in near-lesion areas compared to distant regions (**Figure 3A**). Immunohistochemical staining further confirmed abundant Wnt3a and β-catenin expression in the peripheral regions of fibrotic areas (**Figure 3B**).

### 3.4 Effects of Wnt3a modulation on hepatic echinococcal lesions in mice

**Figure 4:**
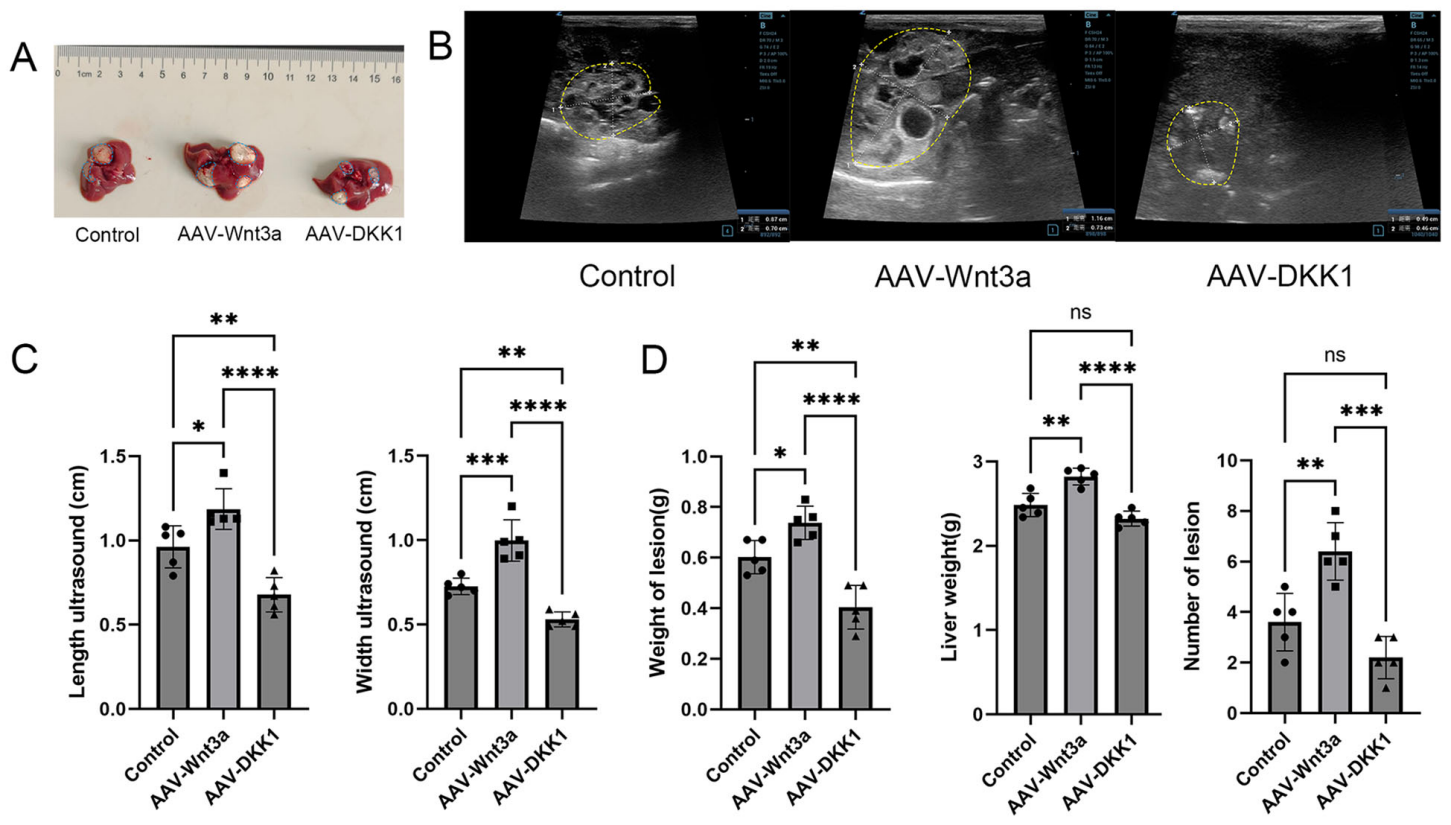
**(A)** Representative images of liver lesions from mice in the control, Wnt3a, and DKK1 groups, with lesion areas outlined by blue dotted lines. **(B)** Ultrasound images of liver lesions from the control, Wnt3a, and DKK1 groups, with lesion boundaries outlined by yellow dotted lines. **(C)** Measurement of maximum and minimum lesion diameters using ultrasound (n = 5). **(D)** Statistical analysis of lesion weight, liver weight, and lesion count (n = 5). Data are presented as mean ± *SD*. *p < 0.05, **p < 0.01, ***p < 0.001, ****p < 0.0001. ns, not significant.

At week 8 post- infection with *E. multilocularis* in mice, liver ultrasound imaging detected one or more hyperechoic signals indicative of hepatic lesions (**Figure 4**). Lesion volumes were significantly smaller in the AAV-DKK1 injection group compared to the AAV-Wnt3a injection group (**Figure 4A**). Post-euthanasia examination of intact liver tissues confirmed that lesions were smaller in the AAV-DKK1 group, while the number of lesions, lesion size, and liver weight were increased in the AAV-Wnt3a group (**Figure 4**).

### 3.5 EMT-MET conversion following Wnt3a modulation

**Figure 5:**
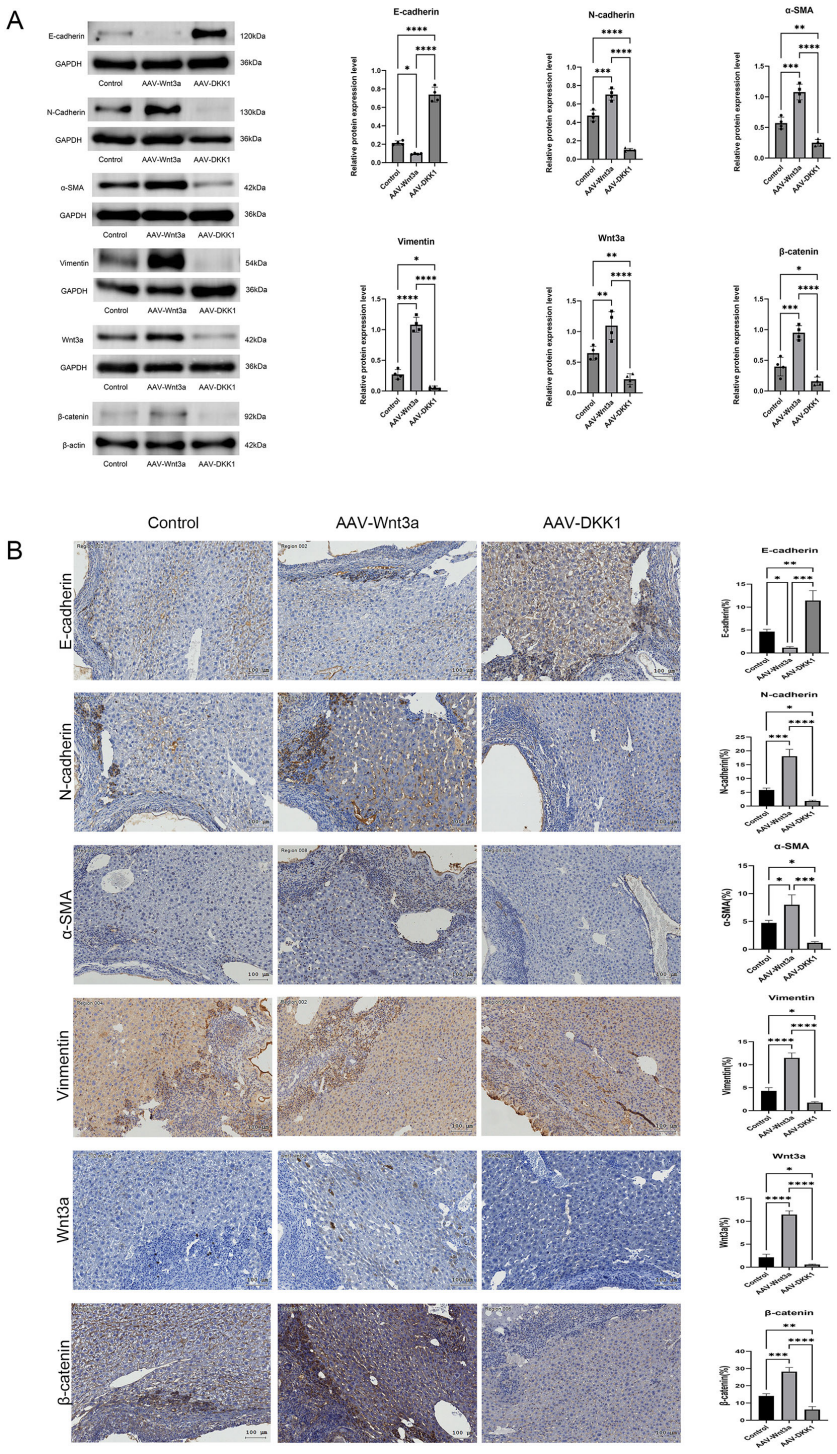
**(A)** Western blot analysis of E-cadherin, N-cadherin, α-SMA, Vimentin, Wnt3a, and β-catenin protein expression, with relative quantification. **(B)** Immunohistochemical staining comparing the expression of E-cadherin, N-cadherin, α-SMA, Vimentin, Wnt3a, and β-catenin among the control, Wnt3a, and DKK1 groups. Scale bar = 100 μm. Data are presented as mean ± *SD*. *p < 0.05, **p < 0.01, ***p < 0.001, ****p < 0.0001.

Model mice were divided into three groups: the control group, the Wnt3a group (Wnt pathway activation group), and the AAV-DKK1 group (Wnt pathway inhibition group). After 8 weeks, pathological staining and western blot analysis of liver tissues revealed that the DKK1 group exhibited the lowest expression levels of β-catenin, Vimentin, N-cadherin, and α-SMA, along with significantly elevated E-cadherin expression. These findings indicate that DKK1 promotes the conversion of EMT to MET in the perilesional areas of AE-induced hepatic fibrosis (**Figure 5**).

In contrast, the Wnt3a group exhibited the highest expression levels of β-catenin, Vimentin, N-cadherin, and α-SMA, accompanied by decreased E-cadherin expression, indicating that Wnt3a enhancement exacerbates AE-induced EMT compared to the control group.

### 3.6 Reduced fibrosis following Wnt3a inhibition

**Figure 6:**
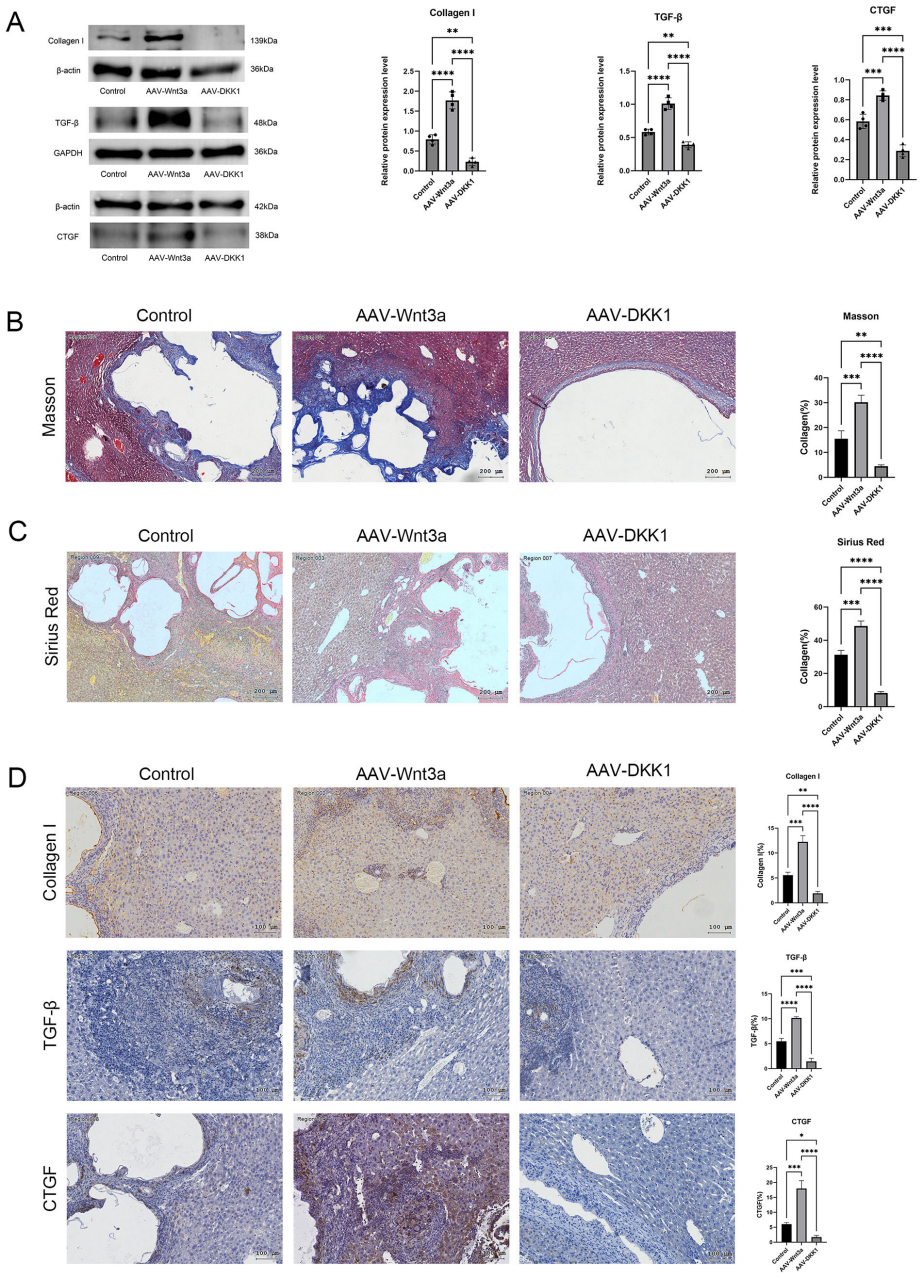
**(A)** Western blot analysis of collagen type I, TGF-β, and CTGF protein expression in liver tissues from control, Wnt3a, and DKK1 groups, with relative quantification. **(B)** Masson’s trichrome staining showing fibrosis in liver tissue from the control, Wnt3a, and DKK1 groups. **(C)** Sirius red staining illustrating fibrosis in liver tissues from the control, Wnt3a, and DKK1 groups. **(D)** Immunohistochemical analysis of collagen type I, TGF-β, and CTGF positive staining areas in liver tissues from control, Wnt3a, and DKK1 groups. Scale bar = 100 μm. Data are presented as mean ± *SD*. *p < 0.05, **p < 0.01, ***p < 0.001, ****p < 0.0001.

Masson’s staining (**Figure 6B**) and Sirius red staining (**Figure 6C**) of mouse liver tissues at 8 weeks post-modeling demonstrated that the DKK1 group exhibited the smallest fibrotic collagen area, whereas the Wnt3a group displayed the most extensive fibrosis. Western blot analysis of Col1a1, TGF-β, and CTGF levels in liver tissues from all three groups (**Figure 6**) showed low collagen expression in the DKK1 group and the highest collagen expression in the Wnt3a group. Immunohistochemical staining further confirmed these findings, showing that Col1a1, TGF-β, and CTGF expression was lowest in the DKK1 group and highest in the Wnt3a group (**Figure 6D**).

## 4 Discussion

Hepatic echinococcosis, also known as hepatic hydatid disease, is a zoonotic parasitic infection prevalent in pastoral regions worldwide (Wen et al., 2019). The disease is primarily classified into two types: cystic echinococcosis (CE), which accounts for the majority of cases, and hepatic AE, a less common but more severe form. Hepatic AE has a poor prognosis, with an untreated 10-year mortality rate exceeding 90%. The disease is characterized by infiltrative growth and hepatic fibrosis surrounding lesions, exhibiting behavior similar to that of malignant tumors, leading to its classification as a “parasitic neoplasm” (Aydinli et al., 2015; Deplazes et al., 2017; Autier et al., 2023). Due to its high recurrence rate following surgical resection, potential for metastasis, and destruction of hepatic parenchymal cells, the development of new therapeutic approaches is imperative to address current treatment limitations.

Hepatic fibrosis is a pathological condition arising from an imbalance between ECM deposition and degradation. It is primarily characterized by abnormal collagen accumulation in the extracellular space. This process may lead to liver cirrhosis and eventual loss of liver function (Parola and Pinzani, 2024). Collagen proteins, the principle structural components of ECM, play a key role in the pathogenesis of hepatic fibrosis (Zhao et al., 2023; Duan et al., 2024). Their expression increases in the early stages of liver fibrosis and continues to rise as the condition progresses. However, hepatic fibrosis remains a potentially reversible condition if the underlying cause is promptly addressed (Baglieri et al., 2021; Xu et al., 2024).

Granulomatous reactions, characterized by fibrous tissue proliferation and infiltration of eosinophils and lymphocytes, are commonly observed around AE lesions, forming characteristic nodules. Pathological examination of patient liver tissues, including H&E, Sirius red, and Masson’s trichrome staining, demonstrated elevated collagen expression in areas surrounding echinococcal lesions. Immunohistochemical staining for collagen type I further confirmed increased expression in these regions. These findings are consistent with previous research by Vuitton and Cao et al. (Vuitton et al., 1986; Cao et al., 2021) which indicate that liver damage induced by AE is closely associated with excessive collagen deposition.

Hepatic injury in AE primarily occurs in regions infiltrated by protoscoleces and adjacent tissues, where it is predominantly characterized by fibroblast proliferation and hepatic fibrosis formation. Therefore, identifying strategies to inhibit or reduce hepatic fibrosis in the early stages of echinococcal infection may be crucial for improving patient prognosis.

TGF-β plays a key role in hepatic fibrosis by activating hepatic stellate cells, a key event in hepatic fibrotic progression (Song Y. et al., 2023). TGF-β is expressed at low levels in both hepatic stellate cells and hepatic parenchymal cells under resting conditions. However, its expression is elevated in chronic hepatitis, where it is predominantly localized to hepatic stellate cells, inflammatory cells, and Kupffer cells, particularly in hepatocytes surrounding fibrotic nodules (Sharkawy et al., 2019). Studies by Song and Link et al. have demonstrated that TGF-β overexpression in hepatocytes exacerbates the progression of hepatic fibrosis (Song J. et al., 2023; Link et al., 2024). Additional research indicates that TGF-β produced by damaged hepatocytes serves as a key signal for hepatic stellate cell activation.

In the present study, TGF-β expression was induced by AE infection and was primarily detected in the perilesional areas of hepatic AE lesions. CTGF, another key factor involved in the activation of hepatic stellate cells and the development of hepatic fibrosis, is produced by various cell types, including hepatocytes, hepatic stellate cells, monocytes, and biliary epithelial cells (Pi et al., 2023). Research by Shen and Sakai demonstrated increased CTGF expression in hepatocytes during hepatic fibrosis induced by bile duct ligation and carbon tetrachloride models (Shen et al., 2017; Sakai et al., 2023). Similarly, studies by Shi and Li et al. demonstrated that hepatocyte-specific overexpression of CTGF contributes to hepatic fibrosis development (Li et al., 2016; Nie et al., 2022).

The findings of the present study indicate that CTGF expression is elevated following human AE infection, with pathological analysis demonstrating significantly increased CTGF expression in regions surrounding echinococcal lesions. This indicates that CTGF may play a key role in promoting hepatic fibrosis in alveolar echinococcal infection. Furthermore, previous studies have shown that hepatocytes significantly upregulate CTGF expression in response to TGF-β stimulation (Chen et al., 2020). These results support the conclusion that both TGF-β and CTGF are key factors driving hepatic fibrosis in AE infection. Following fibrosis inhibition in this study, the expression levels of both TGF-β and CTGF were reduced, consistent with findings from previous studies.

Extensive research on the relationship between tumors and EMT has demonstrated that EMT plays a key role in tumor invasion, metastasis, and chemotherapy resistance. In addition, EMT is recognized as a key process in the progression of hepatic fibrosis. During EMT, epithelial cells acquire mesenchymal characteristics, including increased invasive potential, through the loss of cell junctions, cytoskeletal reorganization, and ECM remodeling (Su et al., 2020). This process is marked by the downregulation of E-cadherin, a protein essential for maintaining cell polarity and morphology, and the upregulation of mesenchymal markers like N-cadherin and α-SMA.

The balance between EMT and MET influences liver repair mechanisms. When EMT predominates, liver repair occurs primarily through fibrosis formation. Conversely, when MET predominates, normal epithelial cell proliferation is enhanced, and fibrosis is reduced (Cicchini et al., 2015). Therefore, inhibiting EMT serves as a potential therapeutic approach for preventing or reversing hepatic fibrosis. HSCs contribute to the progression of hepatic fibrosis by regulating EMT. α-SMA is both a key marker of hepatic fibrosis and a key factor in EMT, HSC activation, and ECM deposition.

In the present study, pathological immunohistochemistry and western blot analysis demonstrated high α-SMA expression in the perilesional regions of AE lesions, indicating the activation of HSCs. Additionally, reduced E-cadherin expression and increased expression of N-cadherin and Vimentin around the lesions were observed, indicating that EMT occurs in the lesion periphery (Pan et al., 2015). Throughout the pathological progression of *E. multilocularis* infection, excessive HSC activation and the subsequent over-deposition of ECM are central to overall pathophysiological changes of the disease.

The Wnt/β-catenin signaling pathway plays a key role in both embryonic development and tumor progression. Increasing evidence supports its involvement in fibrosis across various organs, including the lungs and liver (Liu J. et al., 2022). Previous studies have confirmed the existence of a direct relationship between β-catenin and EMT, and β-catenin is considered a marker for epithelial cells (Syn et al., 2016). Elevated Wnt3a expression has been reported in both carbon tetrachloride (CCl_4_)-induced hepatic fibrosis models and schistosomiasis-induced hepatic fibrosis models in mice. This increase is accompanied by elevated β-catenin and α-SMA expression (Wang et al., 2017; Liu QW. et al., 2022; Wang et al., 2024). Yang et al. demonstrated that inhibition of the Wnt signaling pathway suppresses hepatic stellate cell activity and alleviates hepatic fibrosis, indicating that Wnt/β-catenin signaling plays a role in the progression of hepatic fibrosis (Yang et al., 2017).

In the present study, AAVs carrying either Wnt3a or DKK1 were administered via tail vein injection to *E. multilocularis*-infected mice. Overexpression of Wnt3a in hepatic tissues exacerbated fibrosis, while overexpression of DKK1 reduced the severity of fibrosis. These findings indicate that Wnt signaling plays a significant role in promoting hepatic fibrosis in alveolar echinococcosis. This observation aligns with previous research indicating a positive correlation between Wnt pathway activation and hepatic fibrosis severity. Moreover, DKK1-neutralizing antibody DKN-01 can be safely combined with frontline fluoropyrimidine/oxaliplatin and tislelizumab and demonstrates encouraging activity independent of PD-L1 expression levels. A randomized phase II trial is ongoing in the advanced gastric or gastroesophageal junction adenocarcinoma (Klempner et al., 2025).

### 4.1 Wnt and EMT

The Wnt signaling pathway comprises key components, including Wnt proteins, intracellular β-catenin, glycogen synthase kinase 3 (GSK3), scaffold protein axin, disheveled protein, APC protein, and T-cell factors. The canonical Wnt/β-catenin signaling pathway induces EMT and promotes hepatic fibrosis through β-catenin nuclear translocation and the activation of target gene transcription (Xue et al., 2024). This pathway downregulates E-cadherin expression, thereby accelerating liver fibrosis. The loss of E-cadherin expression is considered a hallmark of EMT (Zhang et al., 2017). Consequently, activation of the Wnt/β-catenin pathway is closely associated with EMT in intrahepatic cells, hepatic stellate cell activation, and the development of hepatic fibrosis.

Findings from this study indicate that the Wnt/β-catenin signaling pathway is involved in both the occurrence of EMT and the formation of fibrosis around hepatic lesions caused by AE. Administration of the Wnt pathway inhibitor DKK1 in AE-infected mice resulted in increased E-cadherin expression and reduced expression of α-SMA, N-cadherin, and Vimentin. These results demonstrate that Wnt pathway inhibition suppresses EMT in the perilesional regions. Additionally, suppression of the Wnt pathway reduced fibrosis in lesion areas compared to the control group, highlighting its potential as a therapeutic target for AE-induced hepatic fibrosis.

Undeniably, there are some limitations to this study. Firstly, the number of surgical specimens and experimental animals was limited, which may have caused bias. Moreover, the regulation of the EMT in fibrosis involves the interaction of multiple signaling pathways, and the clinical significance of inhibiting a single pathway requires further validation. The role of inflammatory cytokines (such as IL-6, IL-1 β, TNF - α) in inhibiting or regulating endogenous DKK1 expression has not been evaluated, and these factors may have an impact on local inflammation progression and fibrosis after AE infection. Furthermore, the research on the molecular mechanism of how the Wnt signaling pathway regulates AE-related fibrosis and EMT is not sufficiently in-depth, lacking exploration of downstream molecular events.

## 5 Conclusion

This study demonstrated that hepatic fibrosis, accompanied by EMT, occurs in perilesional regions following *E. multilocularis* infection in humans. Elevated expression of pro-fibrotic factors, including TGF-β and CTGF, was detected in these regions. Additionally, increased Wnt3a and β-catenin expression around the lesions indicated activation of the Wnt/β-catenin signaling pathway. Similar pathological manifestations were observed in the mouse model of hepatic AE.

Administration of the Wnt pathway inhibitor DKK1 in the mouse model resulted in reduced fibrosis, reversal of EMT, and a decrease in lesion size. These findings provide new insights into potential clinical treatment strategies for hepatic AE and establish a theoretical foundation for further research to determine if DKK1 influences both intrahepatic and distant metastasis of AE.

## Data Availability

The original contributions presented in the study are included in the article/**Supplementary Material**. Further inquiries can be directed to the corresponding author.

## Supplementary material

Supplementary Figure 1 HE staining of human liver with alveolar echinococcosis (AE).

Supplementary Figure 2 Frozen liver sections and small animal imaging after adeno-associated virus (AAV) injections.

## References

[B1] Autier B. Gottstein B. Millon L. Ramharter M. Gruener B. Bresson-Hadni S.. (2023). Alveolar echinococcosis in immunocompromised hosts. Clin. Microbiol. infection 29, 593–599. doi: 10.1016/j.cmi.2022.12.010 36528295

[B2] Aydinli B. Ozturk G. Arslan S. Kantarci M. Tan O. Ahıskalioglu A.. (2015). Liver transplantation for alveolar echinococcosis in an endemic region. Liver Transplant. 21, 1096–1102. doi: 10.1002/lt.24195 26074280

[B3] Baglieri J. Zhang C. Liang S. Liu X. Nishio T. Rosenthal S. B.. (2021). Nondegradable collagen increases liver fibrosis but not hepatocellular carcinoma in mice. Am. J. Pathol. 191, 1564–1579. doi: 10.1016/j.ajpath.2021.05.019 34119473 PMC8406794

[B4] Cao D. Shamsan E. Jiang B. Fan H. Zhang Y. Dehwah M. A. S. (2021). Structural changes and expression of hepatic fibrosis-related proteins in coculture of Echinococcus multilocularis protoscoleces and human hepatic stellate cells. Parasites Vectors 14, 593. doi: 10.1186/s13071-021-05037-1 34857049 PMC8641223

[B5] Chen C. Chen J. Wang Y. Fang L. Guo C. Sang T.. (2023). Ganoderma lucidum polysaccharide inhibits HSC activation and liver fibrosis via targeting inflammation, apoptosis, cell cycle, and ECM-receptor interaction mediated by TGF-β/Smad signaling. Phytomed.: Int. J. phytother. phytopharmacol. 110, 154626. doi: 10.1016/j.phymed.2022.154626 36603342

[B6] Chen P. J. Kuo L. M. Wu Y. H. Chang Y. C. Lai K. H. Hwang T. L. (2020). BAY 41–2272 Attenuates CTGF Expression via sGC/cGMP-Independent Pathway in TGFβ1-Activated Hepatic Stellate Cells. Biomedicines 8, 330. doi: 10.3390/biomedicines8090330 32899801 PMC7554962

[B7] Cicchini C. Amicone L. Alonzi T. Marchetti A. Mancone C. Tripodi M. (2015). Molecular mechanisms controlling the phenotype and the EMT/MET dynamics of hepatocyte. Liver Int. 35, 302–310. doi: 10.1111/liv.12577 24766136 PMC4344819

[B8] Deplazes P. Rinaldi L. Alvarez Rojas C. A. Torgerson P. R. Harandi M. F. Romig T.. (2017). Global distribution of alveolar and cystic echinococcosis. Adv. parasitol. 95, 315–493. doi: 10.1016/bs.apar.2016.11.001 28131365

[B9] Duan B. W. Liu Y. J. Li X. N. Han M. M. Yu H. Y. Hong H. Y.. (2024). An autologous macrophage-based phenotypic transformation-collagen degradation system treating advanced liver fibrosis. Advanced Sci. (Weinheim Baden-Wurttemberg Germany) 11, e2306899. doi: 10.1002/advs.202306899 PMC10870050 38064164

[B10] Klempner S. J. Sonbol M. B. Wainberg Z. A. Uronis H. E. Chiu V. K. Scott A. J.. (2025). DKN-01 in combination with tislelizumab and chemotherapy as first-line therapy in advanced gastric or gastroesophageal junction adenocarcinoma: distinguish. J. Clin. Oncol. 43, 339–349. doi: 10.1200/JCO.24.00410 39432867 PMC11771358

[B11] Kong D. Zhang Z. Chen L. Huang W. Zhang F. Wang L.. (2020). Curcumin blunts epithelial-mesenchymal transition of hepatocytes to alleviate hepatic fibrosis through regulating oxidative stress and autophagy. Redox Biol. 36, 101600. doi: 10.1016/j.redox.2020.101600 32526690 PMC7287144

[B12] Kumar V. Xin X. Ma J. Tan C. Osna N. Mahato R. I. (2021). Therapeutic targets, novel drugs, and delivery systems for diabetes associated NAFLD and liver fibrosis. Advanced Drug delivery Rev. 176, 113888. doi: 10.1016/j.addr.2021.113888 PMC8440458 34314787

[B13] Li S. Lv Y. F. Su H. Q. Zhang Q. N. Wang L. R. Hao Z. M. (2016). A virus-like particle-based connective tissue growth factor vaccine suppresses carbon tetrachloride-induced hepatic fibrosis in mice. Sci. Rep. 6, 32155. doi: 10.1038/srep32155 27562139 PMC4999884

[B14] Li B. Wang L. Qi X. Liu Y. Li J. Lv J.. (2023). NOTCH signaling inhibition after DAPT treatment exacerbates alveolar echinococcosis hepatic fibrosis by blocking M1 and enhancing M2 polarization. FASEB J. 37, e22901. doi: 10.1096/fj.202202033R 37002884

[B15] Link F. Li Y. Zhao J. Munker S. Fan W. Nwosu Z. C.. (2025). ECM1 attenuates hepatic fibrosis by interfering with mediators of latent TGF-β1 activation. Gut. 74(3), 424–439. doi: 10.1136/gutjnl-2024-333213 39448254

[B16] Liu C. Fan H. Guan L. Ge R. L. Ma L. (2021). *In vivo* and *in vitro* efficacy of crocin against Echinococcus multilocularis. Parasites Vectors 14, 364. doi: 10.1186/s13071-021-04866-4 34256821 PMC8278753

[B17] Liu J. Xiao Q. Xiao J. Niu C. Li Y. Zhang X.. (2022). Wnt/β-catenin signalling: function, biological mechanisms, and therapeutic opportunities. Signal transduction targeted Ther. 7, 3. doi: 10.1038/s41392-021-00762-6 PMC8724284 34980884

[B18] Liu Q. W. Ying Y. M. Zhou J. X. Zhang W. J. Liu Z. X. Jia B. B.. (2022). Human amniotic mesenchymal stem cells-derived IGFBP-3, DKK-3, and DKK-1 attenuate liver fibrosis through inhibiting hepatic stellate cell activation by blocking Wnt/β-catenin signaling pathway in mice. Stem Cell Res. Ther. 13, 224. doi: 10.1186/s13287-022-02906-z 35659360 PMC9166579

[B19] Ni X. Yu Q. Li L. (2022). Kinsenoside Protects Against Radiation-Induced Liver Fibrosis via Downregulating Connective Tissue Growth Factor Through TGF-β1 Signaling. Front Pharmaco. 13, 808576. doi: 10.3389/fphar.2022.808576 PMC8814438 35126163

[B20] Pan Q. Wang Y. Q. Li G. M. Duan X. Y. Fan J. G. (2015). Fuzheng huayu recipe ameliorates liver fibrosis by restoring balance between epithelial-to-mesenchymal transition and mesenchymal-to-epithelial transition in hepatic stellate cells. BioMed. Res. Int. 2015, 935903. doi: 10.1155/2015/935903 26881209 PMC4736000

[B21] Parola M. Pinzani M. (2024). Liver fibrosis in NAFLD/NASH: from pathophysiology towards diagnostic and therapeutic strategies. Mol. aspects Med. 95, 101231. doi: 10.1016/j.mam.2023.101231 38056058

[B22] Peters L. Burkert S. Grüner B. (2021). Parasites of the liver - epidemiology, diagnosis and clinical management in the European context. J. hepatol. 75, 202–218. doi: 10.1016/j.jhep.2021.02.015 33636243

[B23] Pi L. Sun C. Jn-Simon N. Basha S. Thomas H. Figueroa V.. (2023). CCN2/CTGF promotes liver fibrosis through crosstalk with the Slit2/Robo signaling. J. Cell communication Signaling 17, 137–150. doi: 10.1007/s12079-022-00713-y PMC10030765 36469291

[B24] Sakai N. Kamimura K. Miyamoto H. Ko M. Nagoya T. Setsu T.. (2023). Letrozole ameliorates liver fibrosis through the inhibition of the CTGF pathway and 17β-hydroxysteroid dehydrogenase 13 expression. J. gastroenterol. 58, 53–68. doi: 10.1007/s00535-022-01929-w 36301364

[B25] Sharkawy R. E. Bayoumi A. Metwally M. Mangia A. Berg T. Romero-Gomez M.. (2019). A variant in the MICA gene is associated with liver fibrosis progression in chronic hepatitis C through TGF-β1 dependent mechanisms. Sci. Rep. 9, 1439. doi: 10.1038/s41598-018-35736-2 30723271 PMC6363805

[B26] Shen K. Feng X. Pan H. Zhang F. Xie H. Zheng S. (2017). Baicalin ameliorates experimental liver cholestasis in mice by modulation of oxidative stress, inflammation, and NRF2 transcription factor. Oxid. Med. Cell. Longevity 2017, 6169128. doi: 10.1155/2017/6169128 PMC5516718 28757911

[B27] Song J. Lv H. Liu B. Hao M. Taylor H. S. Zhang X.. (2023). Let-7 suppresses liver fibrosis by inhibiting hepatocyte apoptosis and TGF-β production. Mol. Metab. 78, 101828. doi: 10.1016/j.molmet.2023.101828 37898449 PMC10641683

[B28] Song Y. Wei J. Li R. Fu R. Han P. Wang H.. (2023). Tyrosine kinase receptor B attenuates liver fibrosis by inhibiting TGF-β/SMAD signaling. Hepatol. (Baltimore Md.) 78, 1433–1447. doi: 10.1097/HEP.0000000000000319 PMC10581422 36800849

[B29] Su J. Morgani S. M. David C. J. Wang Q. Er E. E. Huang Y. H.. (2020). TGF-β orchestrates fibrogenic and developmental EMTs via the RAS effector RREB1. Nature 577, 566–571. doi: 10.1038/s41586-019-1897-5 31915377 PMC7450666

[B30] Syn N. Wang L. Sethi G. Thiery J. P. Goh B. C. (2016). Exosome-mediated metastasis: from epithelial-mesenchymal transition to escape from immunosurveillance. Trends Pharmacol. Sci. 37, 606–617. doi: 10.1016/j.tips.2016.04.006 27157716

[B31] Trampuž S. R. van Riet S. Nordling Å. Ingelman-Sundberg M. (2023). The role of CTGF in liver fibrosis induced in 3D human liver spheroids. Cells 12, 302. doi: 10.3390/cells12020302 36672237 PMC9857203

[B32] Vuitton D. A. Guerret-Stocker S. Carbillet J. P. Mantion G. Miguet J. P. Grimaud J. A. (1986). Collagen immunotyping of the hepatic fibrosis in human alveolar echinococcosis. Z Parasitenkd 72, 97–104. doi: 10.1007/BF00927740 3515794

[B33] Wang F. Chen L. Kong D. Zhang X. Xia S. Liang B.. (2024). Canonical Wnt signaling promotes HSC glycolysis and liver fibrosis through an LDH-A/HIF-1α transcriptional complex. Hepatol. (Baltimore Md.) 79, 606–623. doi: 10.1097/HEP.0000000000000569 PMC10871634 37733267

[B34] Wang Q. Chou X. Guan F. Fang Z. Lu S. Lei J.. (2017). Enhanced wnt signalling in hepatocytes is associated with schistosoma japonicum infection and contributes to liver fibrosis. Sci. Rep. 7, 230. doi: 10.1038/s41598-017-00377-4 28331224 PMC5428310

[B35] Wen H. Vuitton L. Tuxun T. Li J. Vuitton D. A. Zhang W.. (2019). Echinococcosis: advances in the 21st century. Clin. Microbiol. Rev. 32, e00075–e00018. doi: 10.1128/CMR.00075-18 30760475 PMC6431127

[B36] Xu S. Chen Y. Miao J. Li Y. Liu J. Zhang J.. (2024). Esculin inhibits hepatic stellate cell activation and CCl4-induced liver fibrosis by activating the Nrf2/GPX4 signaling pathway. Phytomed.: Int. J. phytother. phytopharmacol. 128, 155465. doi: 10.1016/j.phymed.2024.155465 38471319

[B37] Xue W. Yang L. Chen C. Ashrafizadeh M. Tian Y. Sun R. (2024). Wnt/β-catenin-driven EMT regulation in human cancers. Cell. Mol. Life sciences: CMLS 81, 79. doi: 10.1007/s00018-023-05099-7 38334836 PMC10857981

[B38] Yang Y. Chen X. X. Li W. X. Wu X. Q. Huang C. Xie J.. (2017). EZH2-mediated repression of DKK1 promotes hepatic stellate cell activation and hepatic fibrosis. J. Cell. Mol. Med. 21, 2317–2328. doi: 10.1111/jcmm.13153 28332284 PMC5618695

[B39] Zhang X. Yang M. Shi H. Hu J. Wang Y. Sun Z.. (2017). Reduced E-cadherin facilitates renal cell carcinoma progression by WNT/β-catenin signaling activation. Oncotarget 8, 19566–19576. doi: 10.18632/oncotarget.15361 28223537 PMC5386706

[B40] Zhao Y. Q. Deng X. W. Xu G. Q. Lin J. Lu H. Z. Chen J. (2023). Mechanical homeostasis imbalance in hepatic stellate cells activation and hepatic fibrosis. Front. Mol. Biosci. 10. doi: 10.3389/fmolb.2023.1183808 PMC10157180 37152902

