# Supplementary figures and images for: Activation of the Wnt signaling pathway and its role in epithelial-mesenchymal transition and hepatic fibrosis in alveolar echinococcosis
Source: Front Cell Infect Microbiol. 2025 May 27;15:1583802. doi: 10.3389/fcimb.2025.1583802 (PMC12149095; doi:10.3389/fcimb.2025.1583802)

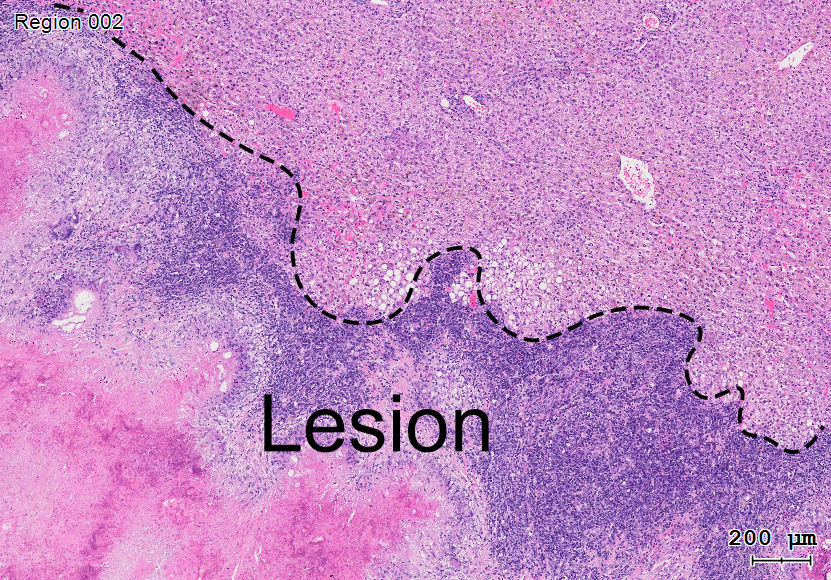

Supplement: Supplementary Figure 1 — HE staining of human liver with alveolar echinococcosis (AE). [file Image1.tif]

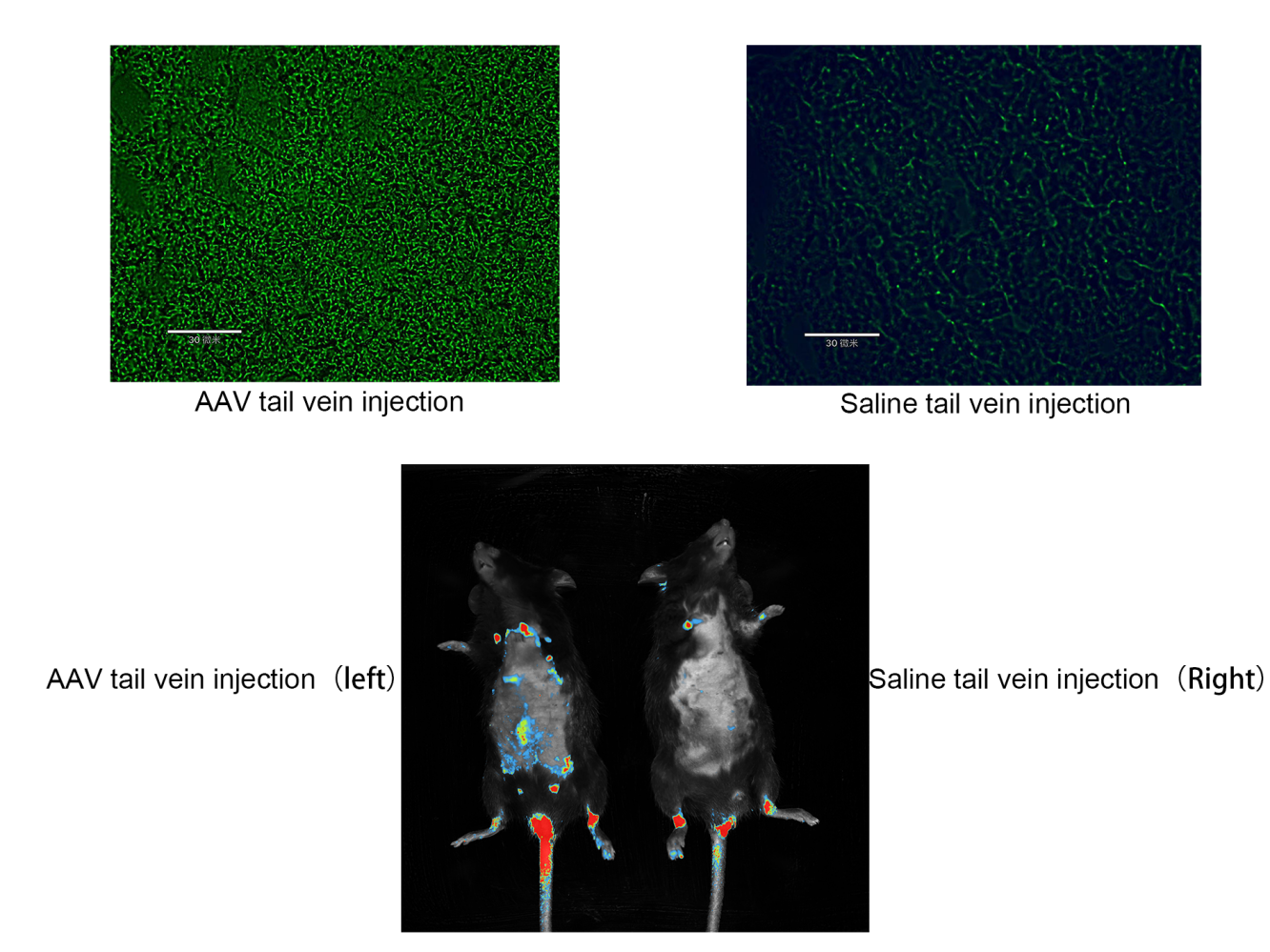

Supplement: Supplementary Figure 2 — Frozen liver sections and small animal imaging after adeno-associated virus (AAV) injections. [file Image2.tif]
