# Supplementary material for: Activation of the Wnt signaling pathway and its role in epithelial-mesenchymal transition and hepatic fibrosis in alveolar echinococcosis
Source: Front Cell Infect Microbiol. 2025 May 27;15:1583802. doi: 10.3389/fcimb.2025.1583802 (PMC12149095; doi:10.3389/fcimb.2025.1583802)
Supplement: Supplementary file 3 [file Table1.docx]

Table 1. The basic information of 20 patients.

| Number | Age | Gender | Date of Operation | Lesion size | Blood type | Nationality |
| --- | --- | --- | --- | --- | --- | --- |
| 1 | 43y | F | 2021/6/2 | 10*8 cm | O | Tibetan nationality |
| 2 | 55y | F | 2021/6/2 | 9*9 cm | B | Mongolian nationality |
| 3 | 34y | M | 2021/6/17 | 7*4 cm | A | Hui nationality |
| 4 | 29y | M | 2021/8/19 | 13*11 cm | O | Mongolian nationality |
| 5 | 21y | M | 2021/9/16 | 8*6 cm | A | Tibetan nationality |
| 6 | 35y | M | 2021/9/16 | 15*13 cm | O | Tibetan nationality |
| 7 | 49y | F | 2021/12/29 | 10*9 cm | AB | Han nationality |
| 8 | 63y | M | 2022/3/3 | 15*10 cm | B | Tibetan nationality |
| 9 | 44y | M | 2022/3/15 | 9*6 cm | A | Salar nationality |
| 10 | 52y | F | 2022/4/29 | 7*5 cm | O | Tibetan nationality |
| 11 | 27y | F | 2022/6/10 | 6*4 cm | B | Tibetan nationality |
| 12 | 40y | F | 2022/6/10 | 10*8 cm | O | Mongolian nationality |
| 13 | 27y | F | 2022/7/27 | 13*10 cm | O | Tibetan nationality |
| 14 | 19y | F | 2022/8/5 | 4*4 cm | A | Tibetan nationality |
| 15 | 32y | M | 2022/12/15 | 5*3 cm | AB | Han nationality |
| 16 | 50y | F | 2022/12/30 | 11*6 cm | AB | Tibetan nationality |
| 17 | 34y | M | 2022/12/30 | 13*8 cm | B | Tu nationality |
| 18 | 49y | F | 2023/1/3 | 9*8 cm | O | Mongolian nationality |
| 19 | 32y | F | 2023/1/10 | 8*4 cm | B | Salar nationality |
| 20 | 62y | M | 2023/2/22 | 7*5 cm | A | Tibetan nationality |
